# HOMA-estimated insulin resistance as an independent prognostic factor in patients with acute pancreatitis

**DOI:** 10.1038/s41598-019-51466-5

**Published:** 2019-10-17

**Authors:** Seung Kook Cho, Ji Hye Huh, Jin Sae Yoo, Jae Woo Kim, Kyong Joo Lee

**Affiliations:** Department of Internal Medicine, Yonsei University Wonju College of Medicine, Wonju, South Korea

**Keywords:** Predictive markers, Gastroenterology

## Abstract

This prospective study investigated the relationship between insulin resistance assessed using the homeostatic model assessment of insulin resistance (HOMA-IR) and the prognosis of acute pancreatitis (AP). A total of 269 patients with AP were recruited in this study. HOMA-IR scores were calculated using fasting insulin and plasma glucose levels. Patients were then categorized into the non-insulin-resistant group (HOMA-IR <2.5) and the insulin-resistant group (HOMA-IR ≥2.5). We performed multivariable logistic regression analysis to investigate the independent association between IR assessed using HOMA-IR and the severity of AP. We also conducted receiver operating characteristic analysis to investigate the predictive ability of HOMA-IR for severe AP. The proportion of patients with severe AP (according to the Atlanta classification) and the percentage of ICU admissions and mortality were higher in patients with insulin resistance than in those without insulin resistance. The area under the curve (AUC) of HOMA-IR for predicting severe AP was 0.719 (95% CI 0.59–0.85, *P* = 0.003). This value was not significantly different from the AUCs of other AP scoring systems such as CTSI, Ranson, and BISAP. Insulin resistance was the only independent factor for either ICU admission (OR 5.95, 95% CI 1.95–18.15, *P* = 0.002) or severe AP (OR 6.72, 95% CI 1.34–33.62, *P* = 0.020). Our findings suggest that the HOMA-IR score is an independent prognostic factor in patients with acute pancreatitis. This finding indicates that insulin resistance is potentially involved in the mechanism for severe AP.

## Introduction

Acute pancreatitis (AP) is an acute inflammatory process in which the pancreatic injury may remain localized, spread to nearby tissues, or lead to systemic inflammation through activation of cytokine cascades. The underlying pathophysiology behind the progression of local pancreatic injury to systemic inflammation has not been fully elucidated^1^. Although most cases of AP are self-limiting, approximately 15% of patients develop serious conditions. Despite improvements in knowledge regarding AP, severe AP (SAP) still has a mortality rate of 2–9%^2,3^.

Recent studies have demonstrated that metabolic abnormalities, such as diabetes, hypertriglyceridemia, morbid obesity, vitamin D deficiency and the apolipoprotein B to A-I ratio, are closely related to the severity and prognosis of AP^4–8^. In line with these findings, blood glucose level, which an indicator of ongoing metabolic dysfunction, is used as a criterion in several AP-severity scoring systems such as the Ranson score and the Glasgow-Imrie criteria.

Insulin resistance is defined clinically in terms of the failure of insulin to maintain glucose homeostasis^9^ and it plays an essential role in the pathogenesis of chronic metabolic disease^10–12^. There has been ample evidence indicating an association between insulin resistance and diabetes, dyslipidemia, and metabolic syndrome (MS), all of which are well-known contributors to the development and severity of acute pancreatitis^4,13–15^. Since insulin resistance is a chronic, low-grade inflammatory status^16^, insulin resistance has been postulated to play a pathogenic role in other inflammatory diseases such as acute pancreatitis. This hypothesis is supported by reports of pre-existing diabetes increasing the risk of AP development and progression to SAP, as well as the risk of local and systemic complications in AP^17,18^. However, although one study reported increased insulin resistance in patients with AP^19^, little is known regarding the association between insulin resistance and the severity of AP. Therefore, the aim of this study was to investigate whether insulin resistance is associated with the prognosis of AP. In the present study, we assessed insulin resistance using the homeostatic model assessment of insulin resistance (HOMA-IR), which is the most widely validated surrogate measure of general insulin resistance^20^.

## Results

### Patient characteristics

Table 1 shows the baseline characteristics of all patients. A total of 269 patients were enrolled with a mean age of 57.6 ± 18.6 years, and 179 (66.5%) of the patients were male. The etiologies for AP included gallstones (47.6%), alcohol (34.6%), hypertriglyceridemia (5.9%), and idiopathic (11.9%). At the initial evaluation, 99 (36.8%) patients had hypertension, 72 (26.8%) had diabetes mellitus, and the mean body mass index (BMI) was 24.2 ± 4.8 kg/m^2^. According to the Atlanta classification, 173 (64.3%) patients were classified into the mild group, 79 (29.4%) into the moderately severe group, and 17 (6.3%) into the severe group. The median Ranson, CTSI, and BISAP scores of the patients were 2, 2 and 1, respectively, and the median hospital stay for the patients was 5 days. Among all patients, 34 (12.6%) were admitted to the ICU, and 4 (1.5%) died during treatment.

**Table 1:** Baseline characteristics of all patients.

Variable	N = 269
Sex (male, female)	179 (66.5%), 90 (33.5%)
Age, years	57.6 ± 18.6
Etiology of acute pancreatitis	
Gallstone	128 (47.6%)
Alcohol	93 (34.6%)
Hypertriglyceridemia	16 (5.9%)
Idiopathic	32 (11.9%)
Smoking	107 (39.8%)
Hypertension	99 (36.8%)
Diabetes Mellitus	72 (26.8%)
Body mass index, kg/m^2^	24.2 ± 4.8
Atlanta classification (2012)	
Mild	173 (64.3%)
Moderately severe	79 (29.4%)
Severe	17 (6.3%)
Ranson (median)	2 (1–4)
CTSI (median)	2 (1–3)
BISAP (median)	1 (1–2)
Hospital stay, days (median)	5 (3–8)
Intensive care unit admission	34 (12.6%)
Mortality	4 (1.5%)
**Laboratory findings**	
C-reactive protein, mg/dL	
On admission	4.5 ± 6.4
After 72 hours	9.2 ± 7.6
Procalcitonin, ng/mL	7.3 ± 24.2
Triglycerides, mg/dL	242.9 ± 687.7
HbA1c, %	6.1 ± 1.3
HOMA-IR	4.5 ± 8.8
HOMA-ß	79.6 ± 107.1

Results are presented as either the mean ± standard deviation or the median.

CTSI, computed tomography severity index; BISAP, Bedside Index for Severity in Acute Pancreatitis; HOMA-IR, homeostasis model assessment of insulin resistance.

In the initial laboratory findings, the mean CRP and procalcitonin levels of the patients were 4.5 ± 6.4 mg/dL and 7.3 ± 24.2 ng/mL, respectively. The mean TG level was 242.9 ± 687.7 mg/dL. The mean HbA1c was 6.1 ± 1.3%, and the mean HOMA-IR and HOMA-ß were 4.5 ± 8.8 and 79.6 ± 107.1, respectively.

### Comparison of parameters according to insulin resistance

We divided the patients into the non-IR group (patients with HOMA-IR scores <2.5) and the IR group (patients with HOMA-IR scores ≥ 2.5) and compared the clinical data between the two groups (Table 2). More females were included in the IR group and the mean BMI was higher in the IR group. Mean TG level and CRP after 72 hours were higher in the IR group compared to the non-IR group. The proportion of severe AP according to Atlanta classification was also higher in the IR group compared to the non-IR group (*P* = 0.021). Additionally, the percentage of ICU admissions and mortality were higher in the IR group.

**Table 2:** The relationship between HOMA-IR score and various clinical parameters.

	HOMA-IR <2.5 (N = 135)	HOMA-IR ≥2.5 (N = 134)	p-value
Age, years	58.1 ± 18.3	57.3 ± 19.1	0.724
Sex (male, female)	98 (72.6%), 37 (27.4%)	81 (60.4%), 53 (39.6%)	0.035
Etiology			0.230
Gallstone	62 (45.9%)	66 (49.3%)	
Alcohol	53 (39.3%)	40 (29.9%)	
Hypertriglyceridemia	5 (3.7%)	11 (8.2%)	
Idiopathic	15 (11.1%)	17 (12.7%)	
Smoking	62 (45.9%)	45 (33.6%)	0.039
BMI (kg/m^2^)	22.6 ± 4.5	25.8 ± 4.8	<0.001
Hypertension	42 (31.1%)	57 (42.5%)	0.052
Diabetes mellitus	30 (22.2%)	42 (31.3%)	0.091
C-reactive protein, mg/dL			
On admission	5.3 ± 6.8	3.8 ± 5.9	0.056
After 72 hours	8.0 ± 6.4	10.4 ± 8.5	0.012
Procalcitonin, ng/mL	6.5 ± 18.7	8.0 ± 28.5	0.663
Triglycerides, mg/dL	132.4 ± 211.5	353.4 ± 938.2	0.009
Atlanta classification			0.021
Mild	91 (67.4%)	82 (61.2%)	
Moderately severe	41 (30.4%)	38 (28.4%)	
Severe	3 (2.2%)	14 (10.4%)	
Scoring systems			
Ranson ≥3	56 (41.5%)	73 (54.5%)	0.033
CTSI ≥3	36 (26.7%)	44 (32.8%)	0.268
BISAP ≥3	17 (12.6%)	23 (17.2%)	0.292
Hospital stay, days	6.0 ± 5.8	7.2 ± 5.4	0.077
ICU admission, n	10 (7.4%)	24 (17.9%)	0.010
Mortality, n	0	4 (3%)	0.043

HOMA-IR, homeostasis model assessment of insulin resistance; BMI, body mass index; CTSI, computed tomography severity index; BISAP, the Bedside Index for Severity in Acute Pancreatitis; ICU, intensive care unit.

### HOMA-IR for predicting severe acute pancreatitis

**Figure 1:**
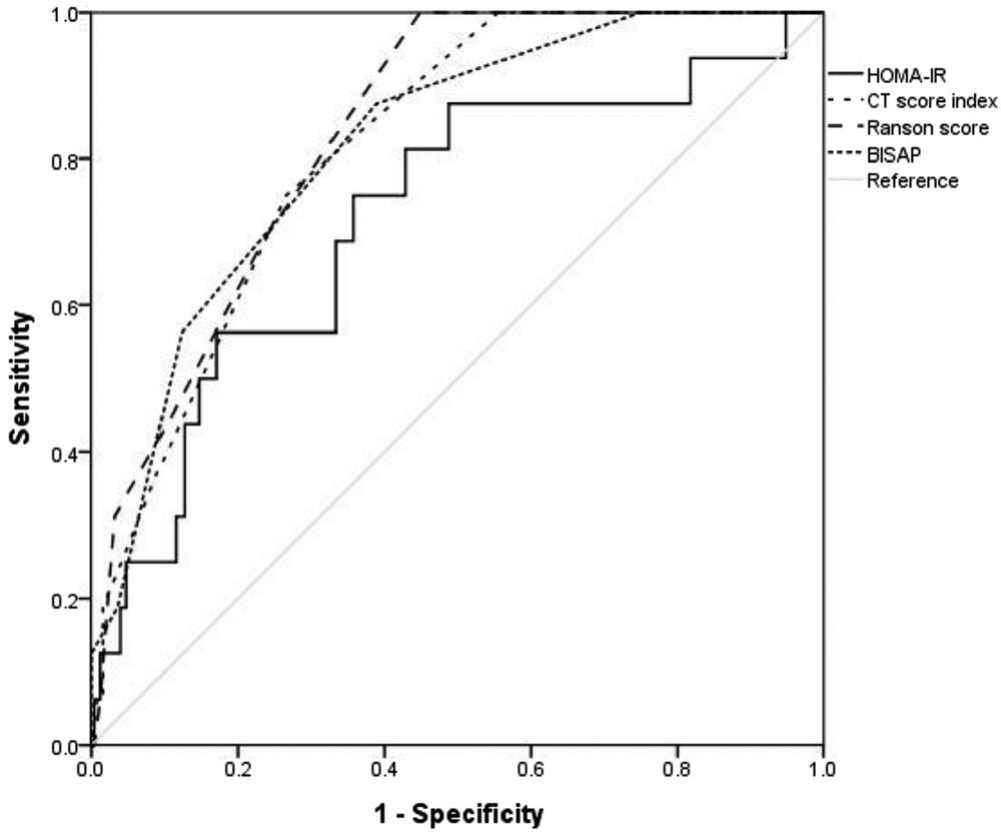
Receiver operator characteristic curve of various factors as predictors of severe acute pancreatitis.

We calculated the area under the curves (AUCs) of HOMA-IR, CTSI, Ranson, and BISAP scores for predicting severe AP using receiver operating characteristic analysis (Table 3). The Ranson score showed the greatest accuracy for prediction of severe AP (AUC = 0.848). The AUC of HOMA-IR for predicting severe AP was 0.719 (95% CI 0.59–0.85, *P* = 0.003). This value was not notable different from the AUCs of the other scoring systems (Fig. 1). We performed a logistic regression analysis to find risk factors predicting severe AP or ICU admission in patients with AP. IR (HOMA-IR ≥2.5) was the only independent factor for ICU admission (OR 5.95, 95% CI 1.95–18.15, *P* = 0.002) or severe AP (OR 6.72, 95% CI 1.34–33.62, *P* = 0.020) (Tables 4 and 5).

**Table 3:** Area under the curve for predicting severe acute pancreatitis.

	AUC	Standard error	95% CI	p-value
HOMA-IR	0.719	0.06	0.59–0.85	0.003
CTSI	0.819	0.04	0.74–0.89	<0.001
Ranson score	0.848	0.03	0.77–0.92	<0.001
BISAP	0.826	0.04	0.74–0.92	<0.001

AUC, area under the curve; CI, confidence of interval; HOMA-IR, homeostasis model assessment of insulin resistance; CT, computed tomography severity index; BISAP, the Bedside Index for Severity in Acute Pancreatitis.

**Table 4:** The association between HOMA-IR and intensive care unit admission.

	OR	p-value^*^	OR	95% CI	p-value^#^
Sex (male)	1.74	0.194	1.48	0.46–4.75	0.515
Age	1.01	0.580	1.04	0.99–1.08	0.148
Gallstone	0.29	0.004	0.48	0.09–2.43	0.378
Alcohol	4.89	<0.001	2.86	0.57–14.31	0.201
Smoking	2.12	0.043	2.17	0.57–8.28	0.257
Hypertension	1.24	0.572	0.99	0.31–3.20	0.995
Diabetes mellitus	1.85	0.110	1.97	0.67–5.81	0.221
Body mass index	0.93	0.040	0.96	0.85–1.08	0.956
C-reactive protein	1.05	0.042	1.01	0.94–1.09	0.763
Procalcitonin	1.02	0.017	1.02	1.00–1.03	0.054
HOMA-IR (≥2.5)	2.73	0.012	5.95	1.95–18.15	0.002

^*^Univariate analysis.

^#^Multivariate analysis.

OR, odds ratio; CI, confidence interval; HOMA-IR, homeostasis model assessment of insulin resistance.

**Table 5:** The association between HOMA-IR and severe acute pancreatitis.

	OR	p^*^	OR	95% CI	p^#^
Sex (male)	0.83	0.724	0.99	0.26–3.86	0.992
Age	1.00	0.920	1.01	0.96–1.05	0.713
Gallstone	0.48	0.181	0.41	0.07–2.37	0.318
Alcohol	1.97	0.192	0.76	0.12–4.87	0.769
Smoking	1.19	0.738	1.96	0.39–9.81	0.411
Hypertension	1.36	0.554	1.18	0.26–5.37	0.834
Diabetes mellitus	0.91	0.869	0.36	0.07–1.99	0.243
Body mass index	1.03	0.569	0.99	0.87–1.14	0.934
C-reactive protein	1.03	0.375	0.98	0.88–1.09	0.702
Procalcitonin	1.01	0.063	1.01	0.99–1.03	0.065
HOMA-IR (≥2.5)	4.73	0.017	6.72	1.34–33.62	0.020

^*^Univariate analysis.

^#^Multivariate analysis.

OR, odds ratio; CI, confidence interval; HOMA-IR, homeostasis model assessment of insulin resistance.

## Discussion

We demonstrated that insulin resistance assessed using HOMA-IR was significantly associated with AP severity and ICU admission. We found that this significant association between insulin resistance and severe AP was independent of the presence of diabetes, the body mass index, and the levels of inflammation markers. We also demonstrated that the ability of insulin resistance to predict severe AP was as good as other traditional scoring systems for AP. These findings indicate that insulin resistance might be critical to the pathogenesis of AP.

An important part of the pathophysiology of AP is inflammation of the pancreatic adipose tissue^13^. MS is a chronic, low-grade inflammatory status characterized by high circulating levels of pro-inflammatory cytokines^16^. The inflammatory changes that accompany MS may intensify both the immune and non-immune responses that can trigger and exacerbate AP^21^, which has been confirmed in several investigations showing an increased incidence and severity of AP in patients with MS^13,14^. The associations among the various components of MS and IR are well documented^11^, and insulin resistance plays an important role in the development of MS^22,23^. However, little is known regarding the association between insulin resistance and the prognosis of AP. Therefore, this study aimed to determine the relationship between insulin resistance and the severity of AP. Insulin resistance is defined as a clinical state of decreased sensitivity or responsiveness to insulin^24^. HOMA-IR has a strong linear correlation with glucose clamp estimates of IR and has been widely used in various prospective clinical trials and clinical research studies^25,26^. A HOMA-IR score ≥2.5 is the accepted cutoff value as an indicator of IR^24,27^. To date, there has been a single study demonstrating IR as a risk factor for post-endoscopic retrograde cholangiopancreatography (ERCP) pancreatitis^28^. HOMA-IR was an independent predictor of post-ERCP pancreatitis and was used as a considerable factor in predicting the risk of post-ERCP pancreatitis and in decreasing related morbidity^28^. However, the authors did not demonstrate an association between IR and the severity of the pancreatitis.

Our study illustrates several important and novel findings. First, more females were included in the IR group, and the mean TG and BMI level were higher, compared to the non-IR group. Hypertriglyceridemia is well known in the etiology of AP, and elevated serum TG is independently and proportionally correlated with persistent organ failure, regardless of etiology^5^. Also, obesity induces a low-grade pro-inflammatory state and is linked with the development of complications in cases of AP^6^. The number of cases with severe AP according to the Atlanta classification was higher in the IR group. Also, the number of ICU admissions and the mortality rate were higher in the IR group compared to the non-IR group. Second, our ROC analysis found that for predicting severe AP, the AUC of HOMA-IR was not significantly different from that of other scoring systems. This implies that a simple measurement of serum chemistries at the clinical baseline may be able to reliably replace traditional prognostic indices that require multiple clinical measurements. Third, IR (HOMA-IR ≥2.5) was the only independent factor for ICU admission or severe AP in our study, but other factors, including DM, BMI, and CRP, were not significantly associated with either the development of severe AP or ICU admission. This result strongly supports the prognostic value of HOMA-IR in patients with AP and is also in line with the results of previous studies that failed to demonstrate an association between DM and the severity of AP^29,30^.

Insulin resistance and hyperglycemia, which are hallmarks of DM, are important factors linked to the susceptibility of diabetics to AP^31^. The existence of links between IR and several pro-inflammatory molecules, such as nuclear factor kB^32^, tumor necrosis factor α^33^, amylin^34^, calcitonin gene-related peptide^35^, leptin^36^ and interleukin-6^37^, has been postulated, and these molecules may play a critical role in the pathogenesis of AP in patients with IR. Also, several *in vitro* studies found that insulin played a protective role against palmitoleic acid-induced AP in rat acinar cells by inhibiting cytosolic calcium overload response^38,39^ and in L-arginine-induced AP rat models by protecting against oxidative stress as well as contributing to acinar cell regeneration^40^. Thus, impaired pancreatic β-cell responsiveness and decreases in circulating insulin caused by pancreatic acinar cell exposure to hyperglycemia, which results in oxidative stress, may play important roles in the susceptibility of diabetics to AP. However, the exact mechanism of the association between IR, DM, and AP has not been fully elucidated. Our findings suggest a possible common pathophysiologic pathway for AP in patients with IR and/or DM. Further investigation into this question may explain the apparently conflicting results regarding a correlation between DM and the incidence and severity of AP presented in past studies.

This study has several limitations. First, the number of patients enrolled in this study was small, and this study was performed in a tertiary care center, which could have resulted in the disproportional inclusion of patients with a severe disease status. Such selection bias might have overestimated the predictive value of HOMA-IR. Second, whether the patients had IR before the diagnosis of AP or IR was a result of AP could not be fully examined. If the patients included in this study would have undergone serum sampling prior to the diagnosis of AP, a more complete explanation as to which is the cause and which is the result may have been available. Third, we did not take into account changes in the HOMA-IR score during treatment, which could have varied according to the progression of AP. Despite these limitations, this study also has a number of strengths. This is the first prospective study investigating the predictive value of HOMA-IR in AP and suggesting HOMA-IR as a possible parameter in improving on previously established severity-scoring systems. Replacing the blood glucose level with HOMA-IR in traditional prognostic scoring systems could improve their performance.

## Conclusions

HOMA-IR, a surrogate marker of insulin resistance, was the only independent prognostic factor for predicting either severe AP or ICU admission in patients with AP. This finding suggests that insulin resistance might influence the risk of SAP irrespective of the cause of the pancreatitis. Therapy targeted at decreasing insulin resistance may have promising role in improving overall outcomes of SAP. Further investigation is needed to confirm the possible deleterious effect of insulin resistance on AP prognosis and to investigate the underlying biological mechanisms for this association in greater detail.

## Methods

### Patients

This was a prospective study of patients with AP in Yonsei University Wonju College of Medicine from March 2015 to April 2018. The study protocol was approved by the International Review Board for Human Research (CR315005-002) of Yonsei University Wonju College of Medicine. This study was performed in accordance with relevant guidelines and regulations. Written informed consent was obtained from all patients.

AP was diagnosed based on the presence of two of the following three features^41^: (1) typical abdominal pain, (2) serum amylase and/or lipase ≥3 times the upper normal limit, and (3) radiologic findings. Laboratory tests on peripheral blood samples, such as hemoglobin, hematocrit, white blood cell count, calcium, phosphorus, blood urea nitrogen, creatinine, lactate dehydrogenase, aspartate aminotransferase, C-reactive protein (CRP, normal range <0.5 mg/dL), and arterial blood gas analysis, were performed at the time of admission. An abdominal computed tomography (CT) scan was performed on all patients upon admission to differentiate AP from other diseases. Once AP was diagnosed, the levels of fasting insulin, glucose, and triglyceride (TG) were verified. The HOMA-IR score was calculated as $\frac{Glucose\times Insulin}{405}$ with glucose in mg/dL, and a HOMA-IR score ≥2.5 was taken as an indicator of IR^24,27^. Additional scoring systems, such as the Ranson score, CT scoring index (CTSI), and BISAP, were applied. The severity of AP was assessed according to the Atlanta 2012 criteria and classified as mild, moderately severe, or severe^41^. Mild AP was defined by the absence of organ failure (OF) and local or systemic complications. Moderately severe AP was defined as transient OF that resolved within 48 hours and was accompanied by local or systemic complications. Severe AP was defined as persistent OF.

### Statistical analysis

All statistical analyses were performed using SPSS software, version 21.0 (SPSS Inc., Chicago, IL, USA). Categorical variables are presented as the frequency and percentage. Continuous variables are presented as either the mean (±standard deviation) or median with range. The paired *t*-test was used to compare continuous variables, and the chi-square test was used to compare categorical variables. The odds ratios (ORs) and confidence intervals (CIs) for having severe AP or an intensive care unit (ICU) admission were calculated using multivariable logistic regression analysis after adjustment for confounding variables. Receiver operating characteristic curves were generated to assess the predictive ability of HOMA-IR for severe AP. A *P*-value < 0.05 was considered statistically significant.

## Data Availability

The datasets analysed for this study are available from the corresponding author upon reasonable request.

## References

[1] Oiva J (2010). Acute pancreatitis with organ dysfunction associates with abnormal blood lymphocyte signaling: controlled laboratory study. Critical Care.

[2] Banks PA (2012). Classification of acute pancreatitis—2012: revision of the Atlanta classification and definitions by international consensus. Gut.

[3] Bradley EL (1993). A clinically based classification system for acute pancreatitis: summary of the International Symposium on Acute Pancreatitis, Atlanta, Ga, September 11 through 13, 1992. Archives of surgery.

[4] Huh JH (2018). Diabetes mellitus is associated with mortality in acute pancreatitis. J Clin Gastroenterol.

[5] Nawaz H (2015). Elevated serum triglycerides are independently associated with persistent organ failure in acute pancreatitis. Am J Gastroenterol.

[6] Krishna SG (2015). Morbid obesity is associated with adverse clinical outcomes in acute pancreatitis: a propensity-matched study. Am J Gastroenterol.

[7] Huh JH, Kim JW, Lee KJ (2019). Vitamin D deficiency predicts severe acute pancreatitis. United European Gastroenterol J.

[8] Huh JH, Jung S, Cho SK, Lee KJ, Kim JW (2018). Predictive value of apolipoprotein B and A-I ratio in severe acute pancreatitis. J Gastroenterol Hepatol.

[9] Haas JT, Biddinger SB (2009). Dissecting the role of insulin resistance in the metabolic syndrome. Current Opinion in Lipidology.

[10] Roberts CK, Hevener AL, Barnard RJ (2013). Metabolic syndrome and insulin resistance: underlying causes and modification by exercise training. Comprehensive Physiology.

[11] Ferrannini E, Haffner SM, Mitchell BD, Stern MP (1991). Hyperinsulinaemia: the key feature of a cardiovascular and metabolic syndrome. Diabetologia.

[12] DeFronzo RA, Ferrannini E (1991). Insulin resistance: a multifaceted syndrome responsible for NIDDM, obesity, hypertension, dyslipidemia, and atherosclerotic cardiovascular disease. Diabetes care.

[13] Mikolasevic I (2016). Metabolic syndrome and acute pancreatitis. European journal of internal medicine.

[14] Szentesi A (2018). Metabolic syndrome elevates the risk for mortality and severity in acute pancreatitis. Pancreatology.

[15] Cho SK, Jung S, Lee KJ, Kim JW (2018). Neutrophil to lymphocyte ratio and platelet to lymphocyte ratio can predict the severity of gallstone pancreatitis. BMC Gastroenterol.

[16] Lee YH, Pratley RE (2005). The evolving role of inflammation in obesity and the metabolic syndrome. Current diabetes reports.

[17] Mikó Alexandra, Farkas Nelli, Garami András, Szabó Imre, Vincze Áron, Veres Gábor, Bajor Judit, Alizadeh Hussain, Rakonczay Zoltán, Vigh Éva, Márta Katalin, Kiss Zoltán, Hegyi Péter, Czakó László (2018). Preexisting Diabetes Elevates Risk of Local and Systemic Complications in Acute Pancreatitis. Pancreas.

[18] Párniczky Andrea, Kui Balázs, Szentesi Andrea, Balázs Anita, Szűcs Ákos, Mosztbacher Dóra, Czimmer József, Sarlós Patrícia, Bajor Judit, Gódi Szilárd, Vincze Áron, Illés Anita, Szabó Imre, Pár Gabriella, Takács Tamás, Czakó László, Szepes Zoltán, Rakonczay Zoltán, Izbéki Ferenc, Gervain Judit, Halász Adrienn, Novák János, Crai Stefan, Hritz István, Góg Csaba, Sümegi János, Golovics Petra, Varga Márta, Bod Barnabás, Hamvas József, Varga-Müller Mónika, Papp Zsuzsanna, Sahin-Tóth Miklós, Hegyi Péter (2016). Prospective, Multicentre, Nationwide Clinical Data from 600 Cases of Acute Pancreatitis. PLOS ONE.

[19] Leśniowski B, Winter K, Kumor A, Małecka-Panas E (2012). Measurement of insulin resistance by HOMA-IR index in patients with acute pancreatitis. Pancreatology.

[20] Bonora E (2002). HOMA-estimated insulin resistance is an independent predictor of cardiovascular disease in type 2 diabetic subjects: prospective data from the Verona Diabetes Complications Study. Diabetes care.

[21] Sawalhi S, Al-Maramhy H, Abdelrahman AI, Allah SEG, Al-Jubori S (2014). Does the presence of obesity and/or metabolic syndrome affect the course of acute pancreatitis?: A prospective study. Pancreas.

[22] Reaven GM (1995). Pathophysiology of insulin resistance in human disease. Physiol Rev.

[23] Grundy SM (1999). Hypertriglyceridemia, insulin resistance, and the metabolic syndrome. Am J Cardiol.

[24] Muniyappa R, Lee S, Chen H, Quon MJ (2008). Current approaches for assessing insulin sensitivity and resistance *in vivo*: advantages, limitations, and appropriate usage. American Journal of Physiology-Endocrinology and Metabolism.

[25] Haffner SM, Miettinen H, Stern MP (1997). The homeostasis model in the San Antonio heart study. Diabetes care.

[26] Wallace TM, Levy JC, Matthews DR (2004). Use and abuse of HOMA modeling. Diabetes care.

[27] Yamada C (2011). Optimal reference interval for homeostasis model assessment of insulin resistance in a Japanese population. Journal of diabetes investigation.

[28] Koksal AR (2016). Insulin resistance as a novel risk factor for post-ERCP pancreatitis: A pilot study. Digestive diseases and sciences.

[29] Shen HN, Lu CL, Li CY (2012). Effect of diabetes mellitus on severity and hospital mortality in patients with acute pancreatitis: A national population-based study. Diabetes care.

[30] Nawaz H, O’Connell M, Papachristou GI, Yadav D (2015). Severity and natural history of acute pancreatitis in diabetic patients. Pancreatology.

[31] Solanki Nicholas S., Barreto Savio G., Saccone Gino T.P. (2012). Acute pancreatitis due to diabetes: The role of hyperglycaemia and insulin resistance. Pancreatology.

[32] Yuan M (2001). Reversal of obesity-and diet-induced insulin resistance with salicylates or targeted disruption of Ikkβ. Science.

[33] Nieto-Vazquez I (2008). Insulin resistance associated to obesity: the link TNF-alpha. Archives of physiology and biochemistry.

[34] DeFronzo RA, Bonadonna RC, Ferrannini E (1992). Pathogenesis of NIDDM: a balanced overview. Diabetes care.

[35] Wick EC (2006). Transient receptor potential vanilloid 1, calcitonin gene-related peptide, and substance P mediate nociception in acute pancreatitis. American Journal of Physiology-Gastrointestinal and Liver Physiology.

[36] German JP (2010). Leptin deficiency causes insulin resistance induced by uncontrolled diabetes. Diabetes.

[37] Pickup JC, Mattock MB, Chusney GD, Burt D (1997). NIDDM as a disease of the innate immune system: association of acute-phase reactants and interleukin-6 with metabolic syndrome X. Diabetologia.

[38] Samad Aysha, James Andrew, Wong James, Mankad Parini, Whitehouse John, Patel Waseema, Alves-Simoes Marta, Siriwardena Ajith K., Bruce Jason I. E. (2014). Insulin Protects Pancreatic Acinar Cells from Palmitoleic Acid-induced Cellular Injury. Journal of Biological Chemistry.

[39] Mankad Parini, James Andrew, Siriwardena Ajith K., Elliott Austin C., Bruce Jason I. E. (2011). Insulin Protects Pancreatic Acinar Cells from Cytosolic Calcium Overload and Inhibition of Plasma Membrane Calcium Pump. Journal of Biological Chemistry.

[40] Hegyi, P. *et al*. Spontaneous and cholecystokinin-octapeptide-promoted regeneration of the pancreas following L-arginine-induced pancreatitis in rat. *Int. J. Pancreatol.* **22**, 193–200, 10.1007/BF02788384 (1997). 10.1007/BF02788384 9444550

[41] Banks PA (2013). Classification of acute pancreatitis–2012: revision of the Atlanta classification and definitions by international consensus. Gut.

